# The Infection of Cucumber (*Cucumis sativus* L.) Roots by *Meloidogyne incognita* Alters the Expression of *Actin-Depolymerizing Factor* (*ADF*) Genes, Particularly in Association with Giant Cell Formation

**DOI:** 10.3389/fpls.2016.01393

**Published:** 2016-09-16

**Authors:** Bin Liu, Xingwang Liu, Ying Liu, Shudan Xue, Yanling Cai, Sen Yang, Mingming Dong, Yaqi Zhang, Huiling Liu, Binyu Zhao, Changhong Qi, Ning Zhu, Huazhong Ren

**Affiliations:** ^1^Key Laboratory of Growth and Developmental Regulation for Protected Vegetable Crops of Beijing, Department of Vegetable Science, College of Horticulture, China Agricultural UniversityBeijing, China; ^2^Changping Agricultural Technology Service CenterBeijing, China

**Keywords:** cucumber, *meloidogyne incognita*, actin-depolymerizing factor (ADF) genes, giant cells, cytoskeleton

## Abstract

Cucumber (*Cucumis sativus* L.) is threatened by substantial yield losses due to the south root-knot nematode (*Meloidogyne incognita*). However, understanding of the molecular mechanisms underlying the process of nematode infection is still limited. In this study, we found that *M. incognita* infection affected the structure of cells in cucumber roots and treatment of the cytoskeleton inhibitor (cytochalasin D) reduced root-knot nematode (RKN) parasitism. It is known that Actin-Depolymerizing Factor (ADF) affects cell structure, as well as the organization of the cytoskeleton. To address the hypothesis that nematode-induced abnormal cell structures and cytoskeletal rearrangements might be mediated by the *ADF* genes, we identified and characterized eight cucumber *ADF* (*CsADF*) genes. Phylogenetic analysis showed that the cucumber *ADF* gene family is grouped into four ancient subclasses. Expression analysis revealed that *CsADF1, CsADF2-1, CsADF2-2, CsADF2-3* (Subclass I), and *CsADF6* (Subclass III) have higher transcript levels than *CsADF7-1, CsADF7-2* (Subclass II genes), and *CsADF5* (Subclass IV) in roots. Members of subclass I genes (*CsADF1, CsADF2-1, CsADF2-2*, and *CsADF2-3*), with the exception of *CsADF2-1*, exhibited a induction of expression in roots 14 days after their inoculation (DAI) with nematodes. However, the expression of subclass II genes (*CsADF7-1* and *CsADF7-2*) showed no significant change after inoculation. The transcript levels of *CsADF6* (Subclass III) showed a specific induction at 21 DAI, while *CsADF5* (Subclass IV) was weakly expressed in roots, but was strongly up-regulated as early as 7 DAI. In addition, treatment of roots with cytochalasin D caused an approximately 2-fold down-regulation of the *CsADF* genes in the treated plants. These results suggest that *CsADF* gene mediated actin dynamics are associated with structural changes in roots as a consequence of *M. incognita* infection.

## Introduction

Root-knot nematodes (*Meloidogyne* spp., RKN) are one of the most destructive pathogens of vegetables, even low nematode levels can cause high yield losses (Mukhtar et al., 2013; Liu et al., 2015). The infective second-stage juveniles (J2s) of nematodes penetrate plant roots and migrate into the vascular cylinder toward the zone of differentiation. The J2s do not kill parasitized cells, but induce the generation of some giant cells as the sole nutritive source by expansion of parenchyma cells in the root vascular tissue (Jones et al., 2013; Molinari et al., 2014). During giant cell expansion, the organization of the actin cytoskeleton is significantly altered and permanent rearranged, showing large numbers of thick actin bundles and cables throughout the cell cortex (de Almeida Engler et al., 2004; Clément et al., 2009). These actin cables within giant cells may be required to guide the vesicle trafficking that is needed for extensive plasma membrane and cell wall biogenesis during their isotropic growth (Favery et al., 2004).

The plant actin cytoskeleton undergoes a striking reorganization in response to internal and external signals and is involved in different cellular processes essential for plant development (Clément et al., 2009). In response to multiple cellular processes, a range of actin binding proteins (ABPs) can dynamically reorganize and remodele the actin cytoskeleton (Ayscough, 1998; Hussey et al., 2006). The turnover of filamentous actins are regulated by members of the actin-depolymerizing factor (ADF) or cofilin family (Staiger et al., 1997; Carlier, 1998; Maciver and Hussey, 2002). The ADF proteins bind G-/F-actin and sever the actin filaments to increase actin turnover (Carlier et al., 1997; Maciver, 1998; Chen et al., 2000; Andrianantoandro and Pollard, 2006; Pavlov et al., 2007).

Cucumber (*Cucumis sativus* L.) is a good source of vitamins, minerals, fiber, and roughage (Mukhtar et al., 2013; Zhang et al., 2014b), which is one of the reasons why it is grown all over the world. However, this popular vegetable is threatened by tremendous yield losses from *Meloidogyne incognita*, a nematode that attacks cucumber roots, inducing giant cell formation (Wehner et al., 1991; Walters et al., 1993; Mukhtar et al., 2013). A clearer understanding of how *M. incognita* affects cucumber roots, the effects of infection and the associated changes in host genes expression would be of considerable value in developing strategies to prevent such attacks; however, to date such research has been limited.

The cucumber genomic sequence that provides an opportunity to study the nature of the structure, organization and expression of the constituent gene families (Huang et al., 2009). In this current study, we identified the cucumber *ADF* family and compared similarities with sequences to the corresponding orthologs in *Arabidopsis thaliana*. We found ample information on the chromosomal locations, genomic structures, and the expression of cucumber *ADF* genes after nematode infection or cytoskeleton inhibitor treatment, which leads us to a better understanding of the relationships between members of the cucumber *ADF* family and nematode infection.

## Materials and methods

### Plant material and cytoskeleton inhibitors treatment

Seeds of cucumber (*C. sativus*) inbred line 3461 (China Agricultural University) were used in the experiments. Cucumber seeds were surface sterilized and sown in a autoclaved soil (50 nutritive soil and 50 % vermiculite) at 26 ± 2°C in a greenhouse. Single cucumber seedlings with two leaves were transplanted into a 15-cm diameter plastic pot filled with steam-sterilized river sand and randomly placed in a greenhouse, provided with a regular regime of 16 h of light/day and temperature maintained between 26 and 30°C. Plants were regularly watered with Hoagland's solution. For the cytochalasin D treatments, a previously reported method was used (de Almeida Engler et al., 2004), with minor modifications. Individual plants were pre-treated on medium with 2 μM cytochalasin D (Sigma, Beijing, China) for 1 day before being transplanted into 15-cm diameter plastic pots, after that 50 ml 0.5 μM cytochalasin D was added every day at 9 am.

### Nematode inoculation and determination of infestation levels

An *M. incognita* race 3 from Institute of Plant Protection, China Academy of Agricultural Sciences population had been reared previously in a glasshouse on susceptible cucumber plants (Liu et al., 2015). The progression of nematode infection is shown in Supplementary Figure 1. Nematode inoculation and determination of infestation levels were as previously described (Bybd et al., 1983; Molinari et al., 2014). Nematodes were distinguished as motile vermiform individuals (J2s), swollen individuals that had become sedentary (third and fourth stages, SJs) and adult females (AFs) (Molinari et al., 2014).

### Paraffin section of roots and galls

Paraffin embedding technique was used to achieved the paraffin section of roots and galls. Cucumber roots and galls at different time were fixed, embedded, and sectioned as previously described (Kerk et al., 2003; Zhang et al., 2013).

### Transmission electron microscopy

Cucumber roots and galls were fixed, embedded, and sectioned as previously described (Liu et al., 2016). Thin cross-sections were made with a UC6I microtome (Leica, Germany) and examined with a JEM-123O scanning transmission electron microscope (JEOL, Japan).

### Sequence alignment and molecular phylogenetic analysis

Sequence information for 11 *A. thaliana ADF* genes were retrieved from previous study (Ruzicka et al., 2007). To assess the corresponding cucumber orthologs, a basic local alignment search tool (BLAST) was performed for each one of the *A.thaliana* ADF protein sequences against the Cucumber Genome Database (http://cucumber.genomics.org.cn/page/cucumber/index.jsp). Those with the highest amino acid percentage identity were selected. Cucumber ADFs were named according to the annotation of the Cucumber Genome Database. The multiple sequence alignment of CsGID1s and related CsGID1 proteins was performed using ClustalW within the MEGA5 software package (Tamura et al., 2011), and the highlighting conserved sequences were drawn by using BoxShade (http://www.ch.embnet.org/software/BOX_form.html). The phylogenetic tree was constructed using the Neighbor-Joining (NJ) method (Saitou and Nei, 1987) with the Poisson model and 1000 bootstrap replicates test by MEGA5 (Liu et al., 2016). The gene numbers corresponding to every CsADF gene in the Cucumber Genome Database are shown in Supplementary Table 1. The PCR primers used for cloning *CsADF* genes are listed in Supplementary Table 2.

### Subcellular localization

The fusion 35S::CsADF2-3::GFP, 35S:: CsADF7-1::GFP, 35S::CsADF6::GFP, 35S::CsADF5:GFP constructs and the control plasmid of 35S::GFP were transformed into onion epidermal cells as previously described (Zhang et al., 2014a). The PCR primers used for cloning *CsADF* genes are listed in Supplementary Table 2.

### RNA isolation and qRT-PCR

Total RNA was extracted from roots or galls using the SV Total RNA Isolation System (Promega, Beijing, China), and cDNA was synthesized using the MultiScribe™ reverse transcriptase kit (Applied Biosystems, Beijing, China). Quantitative real-time RT-PCR (qRT-PCR) was performed as previously described (Liu et al., 2016), The gene specific primers used are listed in Supplementary Table 2.

## Results

### Cloning of *ADF* genes in cucumber

**Figure 1:**
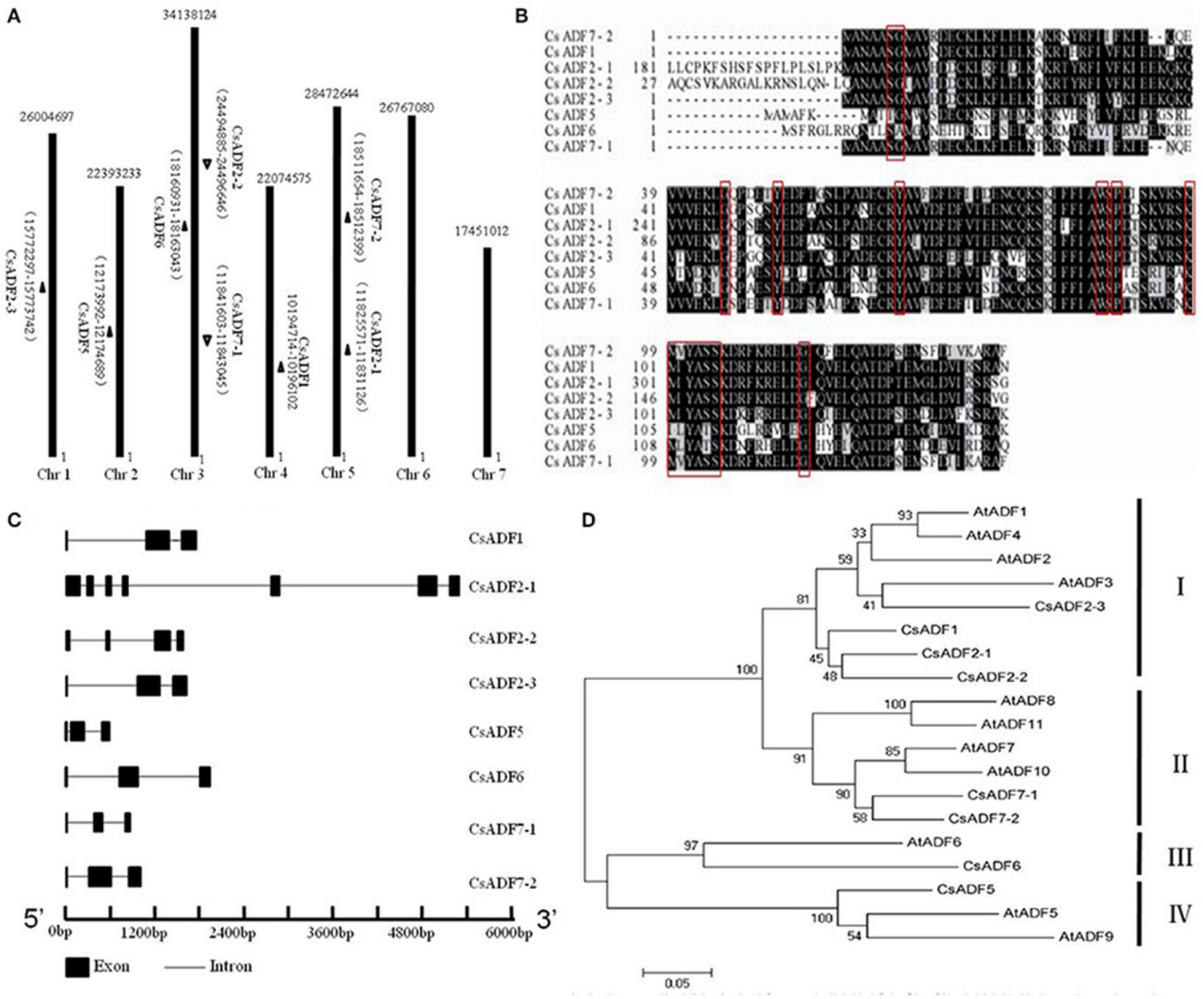
**Cloning of cucumber *ADF* (*CsADF*) genes. (A)** Genomic localization of *CsADF* genes. The arrows next to the gene names show the direction of the scaffold. **(B)** Multiple sequence alignment of eight CsADF proteins. The red boxes indicate the ADF-H domains. **(C)** Genomic organization of *CsADF* genes with exons (bars) and introns (lines). **(D)** Phylogenetic tree of ADF protein sequences from *Arabidopsis thaliana* (AtADF proteins) and cucumber CsADF proteins. Neighbor-joining protein sequence phylogeny showing all 8 ADF proteins from cucumber (*Cs*) and *Arabidopsis* (*At*). Bootstrap values are shown on the branch points of the tree. Subclasses I to IV are indicated by vertical bars on the right.

Using *A.thaliana* ADFs protein sequences, we identified and named eight candidates ADF *genes* based on a BLAST search of the Cucumber Genome Database (http://cucumber.genomics.org.cn/page/cucumber/index.jsp). All *ADF* genes from the cucumber were anchored to cucumber chromosomes, the chromosomal locations and the scaffold sequence directions of the 8 *CsADF* genes were analyzed using BLASTN. Further analysis of the cucumber *ADF* genes revealed that they were distributed on five of the seven cucumber chromosomes and none of the *CsADF* gene was found on chromosomes 6 and 7 (Figure 1A). We observed homologous *CsADF* genes located on different chromosomes in cucumber, suggesting that duplicated events were potentially involved in the evolution of cucumber *CsADFs*.

Previous studies have shown that ADF genes contain a conserved region called the H-domain (Lappalainen et al., 1998), In cucumber, it is present in all *ADF* genes (Figure 1B). As was reported to be the case with *A. thaliana* ADF orthologous genes, all *CsADFs* was predicted to contain three exons and two introns, with the exception of *CsADF2-1* (having seven exons and six introns) *and CsADF2-2* (having four exons and three introns; Figure 1C). Subsequently we further analyzed the phylogenetic relationship with *A. thaliana*. The results revealed that the cucumber ADF gene family is grouped phylogenetically into four ancient subclasses supported by the existence of homologous ADF protein sequences from each subclass in the *A. thaliana*. (Figure 1D). For example, *CsADF6* are significantly more related in protein sequence to *Arabidopsis* ADF6 in subclass III than other cucumber ADF genes.

### Subcellular localization of *CsADF* proteins

**Figure 2:**
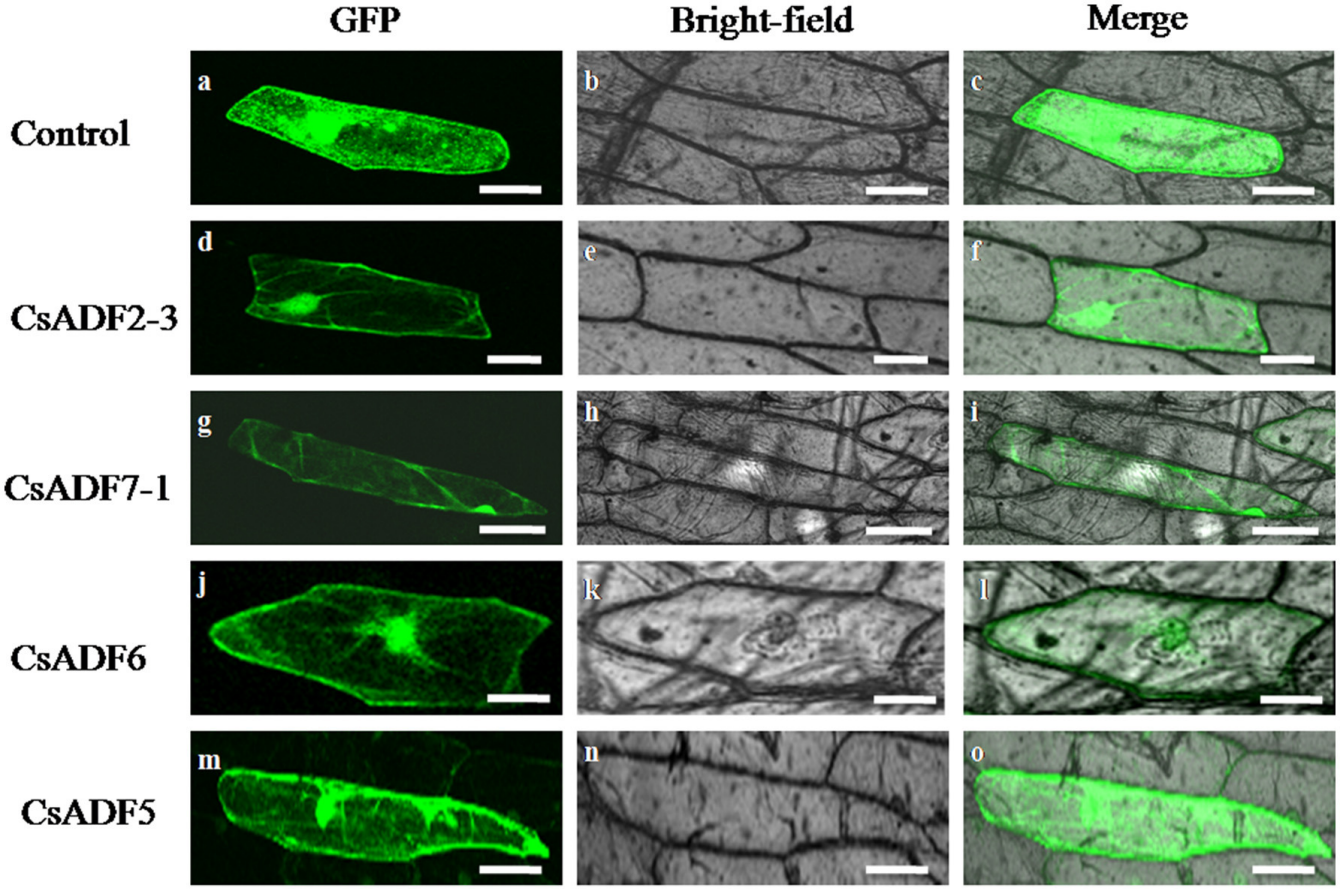
**Subcellular localization of CsADF2-3, CsADF7-1, CsADF5 and CsADF6 in onion epidermal cells**. Green fluorescent protein (GFP) **(A–C)**, CsADF2-3:GFP **(D–F)**, CsADF7-1:GFP **(G–I)**, CsADF5:GFP **(J-L)** or CsADF6:GFP **(M-O)** fusion proteins were transiently expressed in onion epidermal cells under the control of the cauliflower mosaic virus (CaMV) 35S promoter. Bar = 50 μm.

To further determine the subcellular localization of CsADF proteins, sequences corresponding to 35S::CsADF2-3::GFP (subclass I), 35S::CsADF7-1::GFP (subclass II), 35S::CsADF6:GFP (subclass III) and 35S::CsADF5:GFP (subclass IV) fusion proteins were constructed and transiently expressed in onion epidermal using particle bombardment (Zhang et al., 2014a). Transformation with the 35S:GFP control construct resulted in GFP localization throughout the cells (Figures 2A–C), while green fluorescence resulting from expression of each of the four ADF-GFP fusion proteins was localized in both the cytoplasm and the nucleus, with a characteristic filamentous appearance (Figures 2D–O), consistent with a previous report (Tang et al., 2016).

### Expression of *CsADF* genes in *M. incognita* induced galls

**Figure 3:**
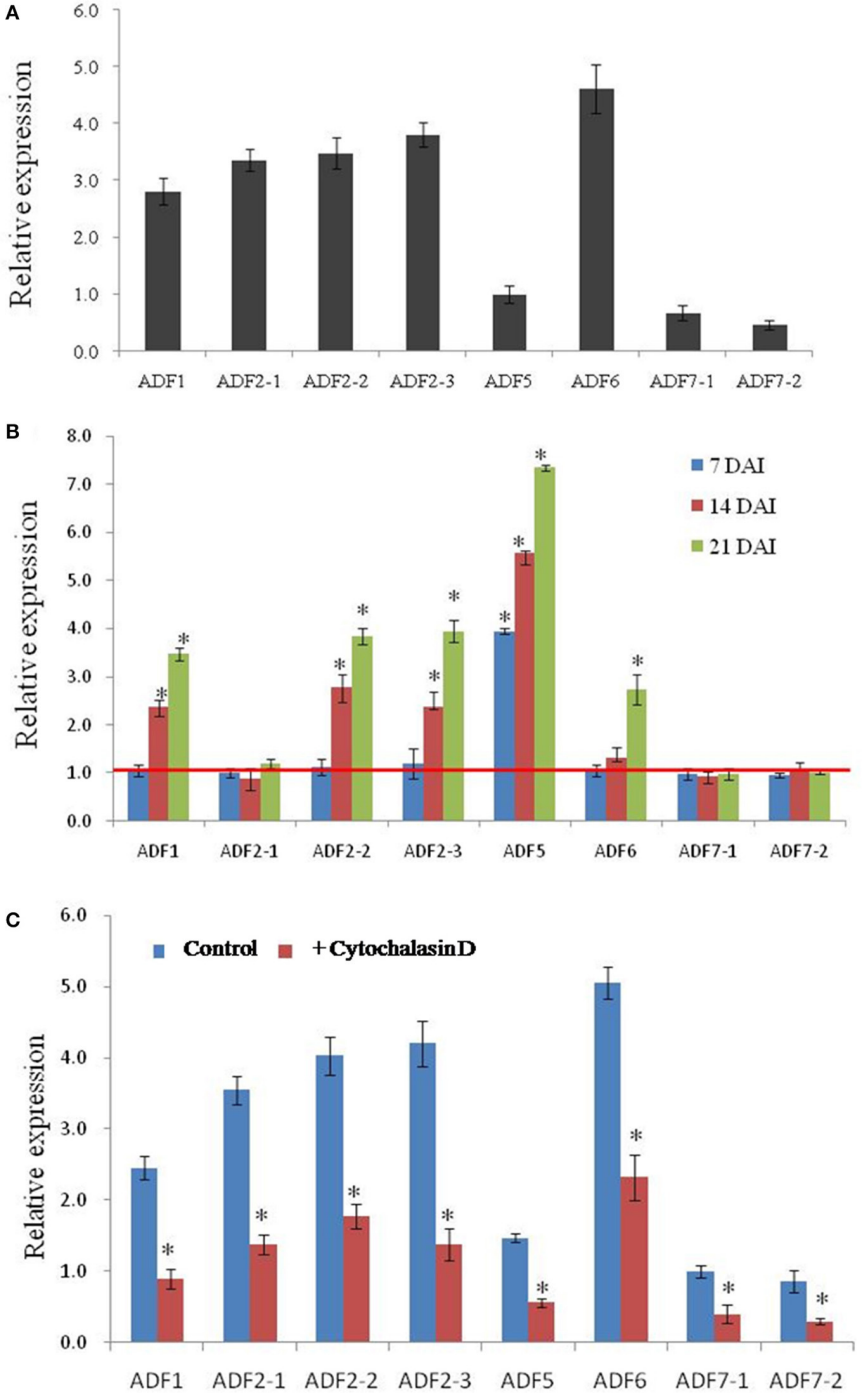
**Analysis of *CsADF* gene expression levels in uninfected roots and galls, as well as in roots, after cytochalasin D treatment. (A)** Quantitative real time-PCR analysis of the transcript abundance of *CsADF* genes in cucumber uninfected roots. **(B)** Relative amount of transcripts of the *CsADF1, CsADF2-1, CsADF2-2, CsADF2-3, CsADF5, CsADF6, CsADF7-1*, and *CsADF7-2* genes in cucumber galls, 7 (blue bars), 14 (red bars), and 21 (green bars) days after infection (DAI) with the nematode, *M. incognita*, compared with uninfected conditions. The ratio was calculated as 2 ^−(ΔCT gene of interest (CT uninfected−CT infected)/Δ CT reference gene(CT uninfected−CT infected)^, where the gene of interest corresponds to one of the analyzed cucumber *ADF* genes and the reference gene is *TUA* (*Csa021066*). The ratio equals 1, meaning that the *ADF* gene expression is not affected by nematode infection. Three biological replicates, represented by three independent quantitative RT-PCR reactions, were performed per sample. The bars (SE) represent mean values of three independent experiments. Statistical significance of the differences between dissected galls and controls (non-meristematic root fragments from non-infected plants) were determined by the Wilcoxon test (*P < 0.05). **(C)** The bars represent ±SD (*n* = 3). Asterisks indicate that the transcripts are significantly down-regulated by cytochalasin D (Wilcoxon test, *P* < 0.05).

Transcript expression analysis by a quantitative real-time reverse transcription-polymerase chain reaction (qRT-PCR) experiment revealed that all *CsADF* genes are expressed in roots. Among them, *CsADF1, CsADF2-1, CsADF2-2, CsADF2-3* (Subclass I genes), and *CsADF6* (Subclass III gene) have a higher expression, while *CsADF7-1, CsADF7-2* (Subclass II genes), and *CsADF5* (Subclass IV gene) have a lower transcript (Figure 3A). To investigate whether *M. incognita* infection correlates with changes in the transcription of ADF genes, we quantified the expression of eight ADF isotypes using qRT-PCR in roots and galls 7, 14, and 21 days after inoculation with 300 *M. incognita* active J2s (DAI; Figure 3B). The transcript levels of five isotypes (*CsADF1, CsADF2-2, CsADF2-3, CsADF5*, and *CsADF6*) were higher in galls of the infected roots compared with those of the control. Members of subclass I genes (*CsADF1, CsADF2-1, CsADF2-2, and CsADF2-3*) except *CsADF2-1* exhibited a specific induction at 14 DAI. The expression of subclass II genes (*CsADF7-1* and *CsADF7-2*) showed no significant change. *CsADF6* (Subclass III gene) showed a specific induction at 21 DAI. It's interesting that *CsADF5* (Subclass IV gene) was weakly expressed in root, but its transcript level was remarkably up-regulated as early as 7 DAI.

### The expression of *CsADF* genes were reduced after cytoskeletal-inhibitor application

Previous study showed that microtubules and actin filaments appeared partially depolymerized throughout the feeding site development (de Almeida Engler et al., 2004), and our results indicated that nematode infection induced the expression of five *CsADF* genes (Figure 3B). Given that cytoskeletal-inhibitor treatments result in the cessation of the development of normal giant cells in *Arabidopsis* (de Almeida Engler et al., 2004), we hypothesized that cytoskeleton inhibitors are likely to reduce the expression of *CsADF* genes. This idea was supported by the qRT-PCR experiment revealing that an approximately 2-fold down-regulation of the *CsADF* genes occurred in roots of treated plants (Figure 3C).

### Treatment of cucumber roots with the cytoskeleton inhibitor cytochalasin D affects cell structure and reduces RKN parasitism

**Figure 4:**
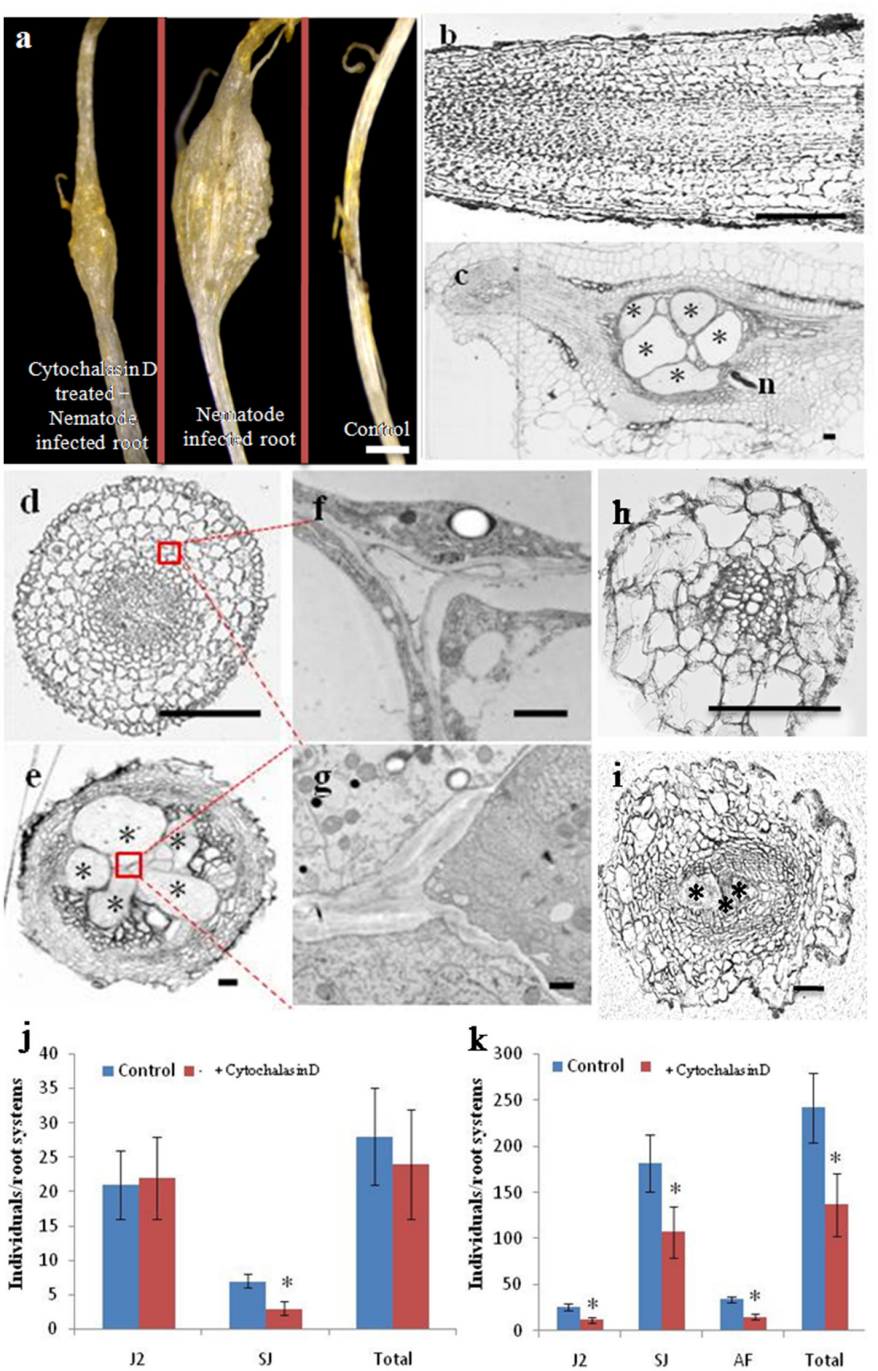
**Cytochalasin D treatment reduces the number of giant cells, as well as RKN parasitism**. Morphological analysis of cucumber roots infected by the nematode *M. incognita.* **(A)** Morphology of cytochalasin D treated and nematode infected, nematode infected, or control roots, Bar = 1 mm. **(B,C)** Longitudinal sections of control and nematode infected roots, Bar = 200 um. **(D,E)** Cross sections of control and nematode infected roots, Bar = 200 um. **(F,G)** Enlarged transmission electron micrographs of the boxes in **(D,E)**, respectively, Bar = 1 um. **(H,I)** Cross sections of control and nematode infected roots in the presence of cytochalasin D, Bar = 200 μm. Samples in **(A–I)** were collected 21 days after infection. Asterisk indicates giant cells. n indicates nematode. **(J,K)** Number of nematodes that penetrated cucumber roots of untreated (Control) and cytochalasin D -treated (+ cytochalasin D) plants, 7 days **(J)** and 21 days **(K)** after inoculation with 300 *M. incognita* invasive juveniles (J2s). Motile juveniles (J2s), sedentary juveniles (SJs) and adult females (AFs) were determined. Values are expressed as averages (obtained from three biological replicates) of the numbers of individuals per root system ±SD (*n* = 20). Asterisks indicate that the means are significantly different, as determined by a *t*-test (*P* < 0.05).

As shown in Figure 4A, infection with *M. incognita* resulted in cucumber root galls; however, in cytochalasin D-treated plantlets, the galls were smaller than in untreated controls. A detailed morphological comparison was performed of nematode infected roots and controls (Figures 4B–I). Cells in both longitudinal and cross sections of control roots were regularly arranged (Figures 4B,D), but in nematode infected root galls, most of the cells were less organized and giant cells were induced at feeding sites (Figures 4C,E). To provide higher resolution of cellular structures, root cells were examined by transmission electron microscopy (TEM) (Figures 4F,G). We focused on variation in the appearance of the cell walls and plasma membrane as several studies have shown that their morphology is particularly important for understanding giant cell development (Jones and Dropkin, 1975, 1976; Jones and Payne, 1978). In the control roots, each cell had a distinct and normal cell wall (Figure 4F), while in the infected roots, ingrowths and degradation occurred on the walls of the giant cells, such that the wall itself was sparse (Figure 4G). The numerous ingrowths greatly increased the surface area of the plasma membrane and were most pronounced adjacent to the vascular tissue, which is a potential site of nutrient influx into giant cells (Baldwin, 1978). Treatment of uninfected roots with cytochalasin D caused cellular defects (Figure 4D), such as a wider root central cylinder and larger cell files. Giant cells in roots at the same stage of infected seedling treated with cytochalasin D were much smaller than those of uninfected and treated roots (Figure 4I) and there were fewer of them than in untreated plants (Figure 4E).

It has been reported that cytochalasin D treatment blocks cytoskeleton dynamics, resulting in the cessation of development of normal giant cell in *Arabidopsis* (de Almeida Engler et al., 2004). We investigated whether cytochalasin D treatment similarly affected RKN parasitism during a compatible interaction with cucumber. Individual plants were pre-treated on media with high concentrations of cytochalasin D (2 μM) for 1 day, after which they were transferred to soil and inoculated with 300 active J2s. Next, 50 ml 0.5 μM cytochalasin D was added every day. Seven and 21 days after inoculation, we counted the number of nematodes that had penetrated the roots (Figures 4J,K). At day 7 after inoculation, there was no significant difference in total nematode number and the numbers of motile invasive forms (J2s) between untreated and cytochalasin D-treated plants (Figure 4J). We observed very few invasive juveniles as they had become sedentary in the roots of untreated plants. However, even fewer sedentary juveniles (SJs) were present in the cytochalasin D treated plants (Figure 4J). Twenty-one days after inoculation, the number of juveniles developing in the roots increased substantially, whereas the presence of motile invasive forms was reduced to a small fraction. However, the roots of cytochalasin D treated plants exhibited a significant reduction in the number of both J2s and SJs compared with untreated plants (Figure 4K). Although very few AFs were present at this stage, no differences were evident between treated and untreated plants. In general, the number of juveniles that penetrated the roots 21 days after inoculation was approximately two-thirds of the number of inoculated juveniles in the untreated plants and one-half of the number of inoculated juveniles in the treated plants.

## Discussion

Although breeders looked for RKN -resistant cucumber species from many years ago, none of cucumber species was found immune or highly resistant to *M. incognita* (Wehner et al., 1991; Walters et al., 1993; Mukhtar et al., 2013). Moreover, molecular mechanism on how nematodes inducing giant cell formation and expansion in cucumber root is still unclear.

Gene expression patterns and protein subcellular localization and can provide important clues as to their function, and so we investigated the subcellular localization of the CsADF proteins. As shown in Figure 2, heterologously expressed GFP proteins fused to CsADF2-3 (subclass I), CsADF7-1 (subclass II), CsADF6 (subclass III) and CsADF5 (subclass IV) were detected in both the cytoplasm and nucleus, in the form of filaments, which is consistent with previous studies of ADF proteins (Tang et al., 2016).

It has been reported that cytoskeletal changes in giant cells are triggered by the depolymerization of F-actin and microtubules (MTs) (Caillaud et al., 2008; Clément et al., 2009), and that this depolymerization may cause a decrease in the viscosity of the cytoplasm (Gross et al., 1993; de Almeida Engler et al., 2004). The actin depolymerizing factor (ADF)/cofilin family has emerged as a central regulator of actin turnover (Augustine et al., 2008). Biochemical studies show that ADF/cofilin is capable of severing and depolymerizing actin filaments from their pointed ends (Carlier et al., 1997; Andrianantoandro and Pollard, 2006)., and the relaxation of the cytoskeleton in nematode feeding sites may open the way for a successful infection (de Almeida Engler et al., 2004). Analysis of the transcription profiles of eight cucumber ADF genes revealed that in the course of infection over 21 days, a specific ADF family expression signature was observed. Quantitative RT-PCR results showed that the transcription of five genes (ADF1, 2–2, 2–3, 5, and 6) was significantly increased in nematode-induced galls (Figure 3B). Out of these five, ADF5 was induced from early stages of infection (7 DAI); ADF1, ADF2–2, and ADF2–3 were induced from the middle stages of infection (14 DAI); ADF6 was induced from the later stages of infection (14–21 DAI). Transcriptional activation of ADF genes suggests that more ADF or cofilin family may be needed to rearrangement the cytoskeleton in the large galls. These results above revealed that *ADF* genes possibly facilitate nematodes feeding in cucumber root.

Cytochalasin D is an alkaloid compound that inhibits globular actin addition and loss from actin filaments, resulting in a fragmented ACT cytoskeleton (Sampath and Pollard, 1991). Studies with *A. thaliana* indicated that extended treatments with low concentrations (0.5 μM) of cytochalasin D do not prevent root growth, and that nematodes are able to complete their life cycle in the treated roots (Sampath and Pollard, 1991; de Almeida Engler et al., 2004). Moreover, it was reported that microtubules and actin filaments appeared to be partially depolymerized throughout the feeding site development (de Almeida Engler et al., 2004), which likely reflects the up-regulated expression of *ADF* genes. Our results indicate that the transcript levels of the *ADF* genes were reduced after cytochalasin D application (Figure 3), suggesting that the application of cytochalasin D and nematode infection had opposite effects on the expression of ADF genes.

Giant cells induced by root knot nematodes are large multinucleated feeding cells containing a dense cytoplasm generated during a complex host-parasite association in plant roots (de Almeida Engler et al., 2004). Our morphological analyses showed that giant cells were induced and most of the cells were less organized in nematodes infected root galls (Figure 4), suggesting that the actin cytoskeleton may be dynamically reorganized and remodeled by *M. incognita* in cucumber roots. In *Arabidopsis*, nematode feeding involves the retrieval of large cytoplasm volumes (Muller et al., 1981), and a degree of cytoskeleton fragmentation may facilitate the suction and ingestion of cytoplasm during the feeding process of nematodes (de Almeida Engler et al., 2004). In addition, de Almeida Engler et al. (2004) also demonstrated that the cytoskeleton rearrangements and depolymerization induced by parasitic nematodes may be essential for a successful feeding process and the development of galls. Moreover, the expression levels of cytoskeletal components were up-regulated to allow the assembly of a new cytoskeleton in expanding feeding cells (de Almeida Engler et al., 2004). The results presented in Figure 4 indicate that the number of giant cells and RKN parasitism were reduced after cytochalasin D application, from which we infer that the application of cytochalasin D reduces RKN parasitism by affecting the giant cell development.

To conclude, we identified eight cucumber *ADF* genes that are homologs of *A. thaliana ADF* genes, and determined that the transcript levels of several *ADF* genes were affected by *M. incognita* infection. In addition, we found that the expression of *CsADF* genes and RKN parasitism were reduced by the application of cytochalasin D. Our results suggest that *CsADF* gene-mediated actin dynamics may be involved in *M. incognita* infection of cucumber roots.

## Author contributions

HR conceived the project, BL designed the research; BL, XL, YL, MD, YZ, HL, BZ, CQ, and NZ performed the experiments; HR, BL, and YL analyzed data; BL drafted the manuscript, XL and HR revised the manuscript, all authors approved the manuscript.

## Funding

This work was supported by National Public Service Sectors (Agriculture) Project of China (201203003), Beijing Agricultural Innovation Consortium (BAIC01-2016) and Agricultural Scientific and Technological Project (20160415) to HR.

### Conflict of interest statement

The authors declare that the research was conducted in the absence of any commercial or financial relationships that could be construed as a potential conflict of interest.

## Supplementary material

Supplementary Table 1 **Sequence accession numbers of GID1 genes**.

Supplementary Table 2 **List of primers and their uses**.

Supplementary Figure 1 **The developmental of nematodes in infected tissues. (A)** Second stage juvenile (J2s) 3 days after inoculation. **(B)** Sedentary juvenile (SJs) nematode 7 days after inoculation. **(C)** Sedentary juvenile (SJs) nematode 14 days after inoculation. **(D)** Adult female nematode 21 days after inoculation. **(E)** Egg masses 30 days after inoculation. Arrows indicate nematodes or egg masses. Bars = 500 μm.
