# Supplementary material for: The Infection of Cucumber (Cucumis sativus L.) Roots by Meloidogyne incognita Alters the Expression of Actin-Depolymerizing Factor (ADF) Genes, Particularly in Association with Giant Cell Formation
Source: Front Plant Sci. 2016 Sep 16;7:1393. doi: 10.3389/fpls.2016.01393 (PMC5025442; doi:10.3389/fpls.2016.01393)
Supplement: Supplementary Table 1 — Sequence accession numbers of GID1 genes. [file Table1.DOC]

Table S1 Sequence accession numbers of GID1 genes

| **Name** | **GeneBank** |
| --- | --- |
| *CsADF1* | Csa010381 |
| *CsADF2-1* | Csa011269 |
| *CsADF2-2* | Csa004759 |
| *CsADF2-3* | Csa019457 |
| *CsADF5* | Csa016829 |
| *CsADF6* | Csa001107 |
| *CsADF7-1* | Csa008842 |
| *CsADF7-2* | Csa003123 |
