# Supplementary material for: The Infection of Cucumber (Cucumis sativus L.) Roots by Meloidogyne incognita Alters the Expression of Actin-Depolymerizing Factor (ADF) Genes, Particularly in Association with Giant Cell Formation
Source: Front Plant Sci. 2016 Sep 16;7:1393. doi: 10.3389/fpls.2016.01393 (PMC5025442; doi:10.3389/fpls.2016.01393)
Supplement: Supplementary Table 2 — List of primers and their uses. [file Table2.DOC]

**Table S2** List of primers and their uses

| Primer | Sequence (5'-3'') | Used for |
| --- | --- | --- |
| *CsADF1*F | ATGGCCAATGCAGCATCAG | Cloning of *CsADF1* |
| *CsADF1*R | TCAGTTGGCACGGCTTCG |
| *CsADF2-1*F | ATGACAATAACGTGTAATGTGATGA | Cloning of *CsADF2-1* |
| *CsADF2-1*R | TCAGCCAGACCGACTTCTGATA |
| *CsADF2-2*F | ATGGTGAGTTCTCTGAAGCTTACTT | Cloning of *CsADF2-2* |
| *CsADF2-2*R | TCAGCCAACTCGGCTTCG |
| *CsADF2-3*F | ATGGCTAACGCGGCATCT | Cloning of *CsADF2-3* |
| *CsADF2-3*R | TCATTTGGCACGGCTTTTAA |
| *CsADF5*F | ATGGCGATGGCTTTCAAAAT | Cloning of *CsADF5* |
| *CsADF5*R | TCATTTTGCTCTGTCCTTGATCA |
| *CsADF6*F | ATGTCGTTCCGAGGCCTCC | Cloning of *CsADF6* |
| *CsADF6*R | TCATTGAGCACGGTCTCTGATC |
| *CsADF7-1*F | ATGGCGAACGCTGCTTCT | Cloning of *CsADF7-1* |
| *CsADF7-1*R | TTAAAATGCTCGAGCCTTGATTA |
| *CsADF7-2*F | ATGGCGAACGCGGCGTCT | Cloning of *CsADF7-2* |
| *CsADF7-2*R | CTAAAATGCACGTGCCTTGAC |
| *CsADF1*S | CAGGAATGGCTGTGAACGA | qPCR of *CsADF1* |
| *CsADF1*A | AGACCAGGCAATGAAGAAG |
| *CsADF2-1*S | ATTTTCTCACTCTTTCTCGC | qPCR of *CsADF2-1* |
| *CsADF2-1*A | TCATCTTGCTTCTCACCTTT |
| *CsADF2*-2S | GCAGCATCGGGAATAGCAGT | qPCR of *CsADF2* |
| *CsADF2-2*A | CTTGAAGAATCAGGGGACCA |
| *CsADF2-3*S | CGGCATCTGGTATGGCTGT | qPCR of *CsADF2* |
| *CsADF2-3*A | TAAACTTGTCCTTTGAACT |
| *CsADF5*S | ATGGAGATGAAATGGAAGAAAGTG | qPCR of *CsADF2* |
| *CsADF5*A | GTCGGTGACCAAGCGATGAAGAAG |
| *CsADF6*S | CGGTATGTGATCTTTAGGGT | qPCR of *CsADF2* |
| *CsADF6*A | AAGTTGTCTTTGGATGTTGC |
| *CsADF7-1*S | GGAATGGCTGTAAGGGATG | qPCR of *CsADF2* |
| *CsADF7-1*A | CTTTTGATGTGTCAGGGGA |
| *CsADF7-2*S | AAATTGAAGTTTTTGGAGC | qPCR of *CsADF2* |
| *CsADF7-2*A | CCTTTGAAATATCTGGTGA |
| *TUA-S* | ACGCTGTTGGTGGTGGTAC | Internal controls in cucumber |
| *TUA-A* | GAGAGGGGTAAACAGTGAATC |
| *CsADF2-3* | TCCCCCGGGATGGCTAACGCGGCATCT | Cloning of *CsADF2-3* for Subcellular localization |
| *CsADF2-3* | CGCGGATCCGTCATTTGGCACGGCTTTTAA |
| *CsADF5* | TCCCCCGGGATGGCGATGGCTTTCAAAAT | Cloning of *CsADF5* for Subcellular localization |
| *CsADF5* | CGCGGATCCGTCATTTTGCTCTGTCCTTGATCA |
| *CsADF6* | TCCCCCGGGATGTCGTTCCGAGGCCTCC | Cloning of *CsADF6* for Subcellular localization |
| *CsADF6* | CGCGGATCCGTCATTGAGCACGGTCTCTGATC |
| *CsADF7-1* | TCCCCCGGGATGGCGAACGCTGCTTCT | Cloning of *CsADF7-1* for Subcellular localization |
| *CsADF7-1* | CGCGGATCCGTTAAAATGCTCGAGCCTTGATTA |
