# Supplementary figures and images for: The Infection of Cucumber (Cucumis sativus L.) Roots by Meloidogyne incognita Alters the Expression of Actin-Depolymerizing Factor (ADF) Genes, Particularly in Association with Giant Cell Formation
Source: Front Plant Sci. 2016 Sep 16;7:1393. doi: 10.3389/fpls.2016.01393 (PMC5025442; doi:10.3389/fpls.2016.01393)

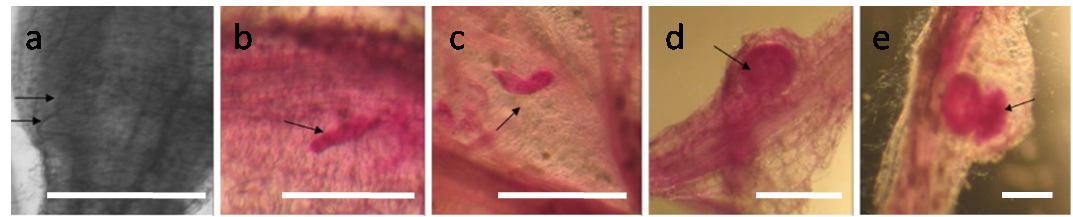

Supplement: Supplementary Figure 1 — The developmental of nematodes in infected tissues. (A) Second stage juvenile (J2s) 3 days after inoculation. (B) Sedentary juvenile (SJs) nematode 7 days after inoculation. (C) Sedentary juvenile (SJs) nematode 14 days after inoculation. (D) Adult female nematode 21 days after inoculation. (E) Egg masses 30 days after inoculation. Arrows indicate nematodes or egg masses. Bars = 500 μm. [file Image1.png]
